# Asymptomatic Middle East Respiratory Syndrome coronavirus infection using a serologic survey in Korea

**DOI:** 10.4178/epih.e2018014

**Published:** 2018-04-15

**Authors:** Yeong-jun Song, Jeong-Sun Yang, Hee Jung Yoon, Hae-Sung Nam, Soon Young Lee, Hae-Kwan Cheong, Woo-Jung Park, Sung Han Park, Bo Youl Choi, Sung Soon Kim, Moran Ki

**Affiliations:** 1 Department of Cancer Control and Population Health, Graduate School of Cancer Science and Policy, National Cancer Center, Goyang, Korea; 2 Korea National Institute of Health, Korea Centers for Disease Control and Prevention, Cheongju, Korea; 3 Korean Society of Infectious Diseases, Seoul, Korea; 4 Department of Preventive Medicine and Public Health, Chungnam National University School of Medicine, Daejeon, Korea; 5 Department of Preventive Medicine and Public Health, Ajou University School of Medicine, Suwon, Korea; 6 Department of Social and Preventive Medicine, Sungkyunkwan University School of Medicine, Suwon, Korea; 7 Department of Preventive Medicine, Hanyang University College of Medicine, Seoul, Korea

**Keywords:** Asymptomatic infection, Epidemiology, Middle East Respiratory Syndrome coronavirus, Nosocomial infections, Outbreak, Enzyme-linked immunespecific assay

## Abstract

**OBJECTIVES:**

The rates of asymptomatic infection with Middle East Respiratory Syndrome (MERS) coronavirus vary. A serologic study was conducted to determine the asymptomatic MERS infection rate in healthcare workers and non-healthcare workers by exposure status.

**METHODS:**

Study participants were selected from contacts of MERS patients based on a priority system in 4 regions strongly affected by the 2015 MERS outbreak. A sero-epidemiological survey was performed in 1,610 contacts (average duration from exposure to test, 4.8 months), and the collected sera were tested using an enzyme-linked immunespecific assay (ELISA), immunofluorescence assay (IFA), and plaque reduction neutralization antibody test (PRNT). Among the 1,610 contacts, there were 7 ELISA-positive cases, of which 1 exhibited positive IFA and PRNT results.

**RESULTS:**

The asymptomatic infection rate was 0.060% (95% confidence interval, 0.002 to 0.346). The asymptomatic MERS case was a patient who had been hospitalized with patient zero on the same floor of the hospital at the same time. The case was quarantined at home for 2 weeks after discharge, and had underlying diseases, including hypertension, angina, and degenerative arthritis.

**CONCLUSIONS:**

The asymptomatic infection was acquired via healthcare-associated transmission. Thus, it is necessary to extend serologic studies to include inpatient contacts who have no symptoms.

## INTRODUCTION

Middle East Respiratory Syndrome (MERS) is a severe respiratory infection caused by a novel beta coronavirus (MERS-CoV) [1-3]. The symptoms of MERS include fever, chills, cough, shortness of breath, gastrointestinal symptoms, expectoration, wheezing, chest pain, hemoptysis, sore throat, headache, myalgia, abdominal pain, vomiting, and diarrhea; it can also cause death in severe cases [3-6].

The causative pathogen of MERS is transmitted via 4 modes: animal-to-human, intra-familial, healthcare-associated, and travel-related [7,8]. The 186 cases that occurred in South Korea (hereafter Korea) were predominantly caused by healthcare-associated transmission [7,9-11], followed by intra-familial transmission.

According to data reported to the World Health Organization, the rates of asymptomatic or mild infection were 44 of 398 (28.60%) in Saudi Arabia, the United Arab Emirates, and the Islamic Republic of Iran between April and June 2014, and 32 of 113 (28.31%) in Saudi Arabia in June 2014 [12,13]. However, Oboho et al. [14] reported that 78.79% (26 of 33) of initially reported asymptomatic patients had at least 1 symptom. In Korea, among the 186 confirmed cases, 3 asymptomatic cases were detected among healthcare workers via screening tests (1.61%) [15]. In serologic studies using indirect immunofluorescence tests for healthcare workers who were at MERS-affected hospitals, 2 of 457 (0.44%) had positive results [16]. However, no report has been published regarding the asymptomatic infection rate among non-healthcare workers in Korea. There is a considerable chance of human-to-human transmission, as well as direct infection via the dromedary camel [17-19]. Therefore, it is necessary to identify the rate of asymptomatic MERS infections in healthcare workers and non-healthcare workers.

## MATERIALS AND METHODS

### Selection and participation of individuals

**Figure 1.**
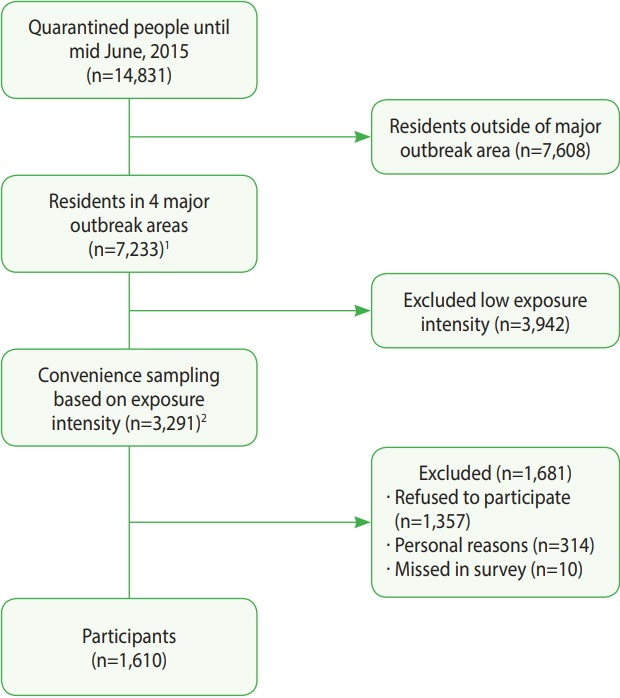
Flowchart of participants in the Middle East Respiratory Syndrome (MERS) serologic survey in Korea. ^1^ Major MERS outbreak areas including Seoul, Gyeonggi, Chungcheong, and Jeonbuk in Korea. ^2^ Selection rates were different by characteristics of exposed MERS case and status of subjects (see the Table 1).

This survey was conducted between August 2015 and February 2016 after the last MERS case diagnosed in July 5, 2015. Based on a database of quarantined individuals provided by the Department of Epidemiologic Investigation of the Korea Centers for Disease Control and Prevention (KCDC), individuals from 4 regions with major outbreaks—Seoul, Gyeonggi, Chungcheong, and Jeonbuk —were selected. Individuals whose MERS status was diagnosed as positive using a polymerase chain reaction test were excluded from the analysis of this study. From the 14,831 quarantined individuals, 7,233 residents (48.8%) living in the 4 major MERS outbreak regions were selected. Of these individuals, calls requesting participation in this study were made to 3,291 individuals (45.5%) according to prioritization groupings. A total of 1,610 individuals (48.9%) ultimately participated in the study (Figure 1). Those who refused to participate have been described in another study [20].

The study individuals were prioritized in groups according to the transmission intensity of the MERS case they were exposed to, as follows: contact with super-spreading events (5 or more individuals infected) [21], contact with spreaders who infected 1 to 4 individuals, and contact with non-spreaders. We selected study subjects according to this prioritization of groups, and the selection rates were 48.8, 16.4, and 15.8%, respectively. We also categorized the subjects according to their exposure intensity (i.e., status when they were exposed to the MERS case), as follows: inpatients or outpatients at a MERS-affected hospital, cohabiting family members or paid caregivers of the MERS case, visitors of the hospitalized MERS case, healthcare workers employed at a MERS-affected hospital, and colleague of the MERS case. We selected more subjects from the categories of family, patients, and visitors (Table 1).

### Study performance

Only individuals who consented to participate in the study through a telephone call and gave written consent at the time of study initiation underwent surveys at local public health centers. Each participant responded to a questionnaire on exposure and provided a blood sample. Following the first serologic test by an enzymelinked immunospecific assay (ELISA), positive or borderline cases were tested using an immunofluorescence assay (IFA) and a plaque reduction neutralization antibody test (PRNT).

### Antibody tests

MERS-CoV antibody levels in all sera collected from contacts were measured using a recombinant S1 protein-coated human anti-MERS-CoV (immunoglobulin G [IgG]) ELISA kit (Euroimmun, Luebeck, Germany), which was used according to the manufacturer’s protocol. The results of the tested samples were determined by calculating the ratio of the optical density (OD) value of the sample to the OD value of the calibrator. Ratios ≥ 1.1 were considered positive, ratios ≥ 0.8 to < 1.1 were considered borderline, and ratios < 0.8 were considered negative.

IFA slides were coated with MERS-CoV non-infected or infected Vero cells. Serum samples were diluted in phosphate-buffered saline (PBS) to 1:10, 1:100, 1:250, and 1:1,000, transferred to IFA slides, and incubated for 30 minutes at 37°C in a humidified chamber. The IFA slides were washed 3 times in PBS-T for 5 minutes each and incubated with fluorescein isothiocyanate-conjugated rabbit anti-human IgG (Abcam, Cambridge, MA, USA) diluted at 1:800 for 30 minutes at 37°C in a humidified chamber. After washing 3 times in PBS-T for 5 minutes, the slides were embedded with a mounting fluid, topped with a cover glass, and observed under a fluorescent microscope.

The neutralizing antibodies in the serum samples were measured by PRNT. The PRNT procedures were performed as follows. In brief, 1:10 diluted sera were heat-inactivated at 56°C for 30 minutes. Heat-inactivated sera were diluted serially 4-fold. After an equal volume of virus (MERS-CoV/KOR/KNIH/002_05_2015) was added to the volume of serum dilutions, these mixtures were incubated at 37°C for 2 hours. The mixtures were added to each well of the 24-well plate cultured with Vero cells. The plate was incubated at 37°C for 60 minutes, and 1 mL of 1.5% carboxymethylcellulose overlay medium was then added. After incubation for 3-4 days, cell staining was performed by crystal violet. The titer of neutralizing antibody by PRNT_50_ was calculated using the Kärber formula, as described previously [22]. A titer above 1:20 was interpreted as positive. All sera with a positive or borderline reaction in ELISA were tested by the IFA and PRNT assays for confirmation, and cases with positive results from any 2 assays were considered to be anti-MERS positive.

This study received approval from the bioethics committee of the KCDC (2015-08-EXP-03-P-A) and the institutional review board of the National Cancer Center (NCC 2016-0058). Informed consent was obtained from study participants or their parent or legal guardian for children under 14.

## RESULTS

Among the 3,291 selected individuals, the response rates of contacts with super-spreading events (5 or more individuals infected), contacts with spreaders who infected 1-4 individuals, and contacts with non-spreaders were 39.8% (552 of 1,388), 40.3% (139 of 345), and 59.0% (919 of 1,558), respectively. According to their status when they were exposed to the MERS case, the highest response rates were found for healthcare workers (99.4%), and family members (97.1%), followed by visitors (75.8%), colleagues (49.0%), and patients (36.8%) (Table 1).

The seropositive rate using ELISA was 1.05% (6 of 574) in patients, 0.33% (1 of 307) in healthcare workers, and 0.43% (7 of 1,610) overall. Among the 7 ELISA-positive individuals, 3 had contact with a super-spreading event (patient zero, case #1) whoinfected 28 individuals [23], 1 had contact with a spreader (case #118) who infected 2 individuals, and 3 had contact with a non-spreader (case #89). Among the ELISA-positive individuals, only 1 was both IFA-positive and PRNT-positive. Therefore, the confirmed rates of asymptomatic MERS infection were 0.17% in patients and 0.060% (95% confidence interval, 0.002 to 0.346) overall (Table 1).

The confirmed asymptomatic case, patient zero, and 11 secondary MERS patients were hospitalized on the same floor of the hospital at the same time [23,24]. The asymptomatic case was quarantined at home for 2 weeks after discharge. The case had underlying diseases that included hypertension, angina, and degenerative arthritis. The case reported no fever, cough, myalgia, or gastrointestinal symptoms during hospitalization or quarantine (Table 2).

## DISCUSSION

Limited information exists regarding MERS-CoV seroprevalence among populations other than confirmed MERS cases. Saudi Arabian data showed that the seroprevalence of MERS-CoV IgG among the general population was 0.15% [25], suggesting that a number of cases of asymptomatic or mild infections may be present in the general population.

Despite a high prevalence of 186 confirmed MERS cases during the outbreak of MERS in Korea, the rate of asymptomatic infection (1.60%) [15] was lower than expected. The rates of asymptomatic infection confirmed using IFA and PRNT in the present study were 0.06% (1 of 1,610) for all contacts and 0.17% (1 of 574) for patients. These results are markedly lower than the rates of 0.27% (2 of 737) among healthcare workers, and 0.44% (2 of 457) among healthcare workers at MERS-affected hospitals in Korea [16]. Moreover, the rate of asymptomatic or mild infection in Saudi Arabia, the United Arab Emirates, and the Islamic Republic of Iran was approximately 28.00% [12,13,18].

The confirmed asymptomatic case presented in this study was a patient at the same hospital as confirmed MERS cases and, unlike in previous studies, was neither an intra-familial infection nor a pediatric infection [26,27]. The low rate of asymptomatic infection in Korea is attributable to the different transmission pathway of MERS infections compared to the Middle East. In Korea, most of the MERS cases were healthcare-associated infections, and none were from an animal. The low asymptomatic infection rate is also attributable to the extensive epidemiological investigation conducted in Korea, including close monitoring of contacts with MERS patients; this helped identify almost all MERS patients. This may also have been attributable to the promotion of proactive identification of patients via mass media and the establishment of communication networks by the government, leading to voluntary reports by people, active quarantine, and countermeasures to this public health crisis [10,28,29].

ELISA is appropriate as a screening tool, as it is 10-fold more sensitive than IFA. However, it may cross-react with seasonal human coronavirus antibodies, so a spike protein-specific IFA is required for confirmation. PRNT is a definitive test when ELISA and IFA have inconclusive results [25]. Only 10% of ELISA-positive results are positive on a neutralization assay [25]. In our study, 1 of 7 patients with borderline or positive ELISA results also had positive results in PRNT. Therefore, the results obtained in our study are accurate because ELISA, IFA, and PRNT were used.

A previous study predicted the pandemic potential of MERS-CoV to be ≤ 5%; however, this does not indicate that the risk has abated [30]. Prerequisites for reducing the risk include improved surveillance, active contact tracing, and the initiation of animal host searching [30,31]. During the outbreak in Korea, MERS was classified as a notifiable infectious disease and was subjected to surveillance [32]. Since MERS is an imported disease in Korea, it is recommended that precautions be taken before travel and that the time of returning from travel and incubation period be considered.

This study showed a low seropositivity in the population of individuals quarantined due to contact with MERS cases. However, there is a possibility that the seropositivity rate was underestimated for the following reasons. Firstly, the participants of the present study were mostly non-healthcare workers and were relatively healthy. Thus, the risk of infection was low. Secondly, the overall participation rate was 48.9%, whereas it was 36.8% in the patient group at a higher risk of infection. Moreover, we only surveyed 10.9% of the quarantined individuals. Therefore, the actual rate of asymptomatic infections may be higher than reported in the present study. Lastly, the present study may have been conducted too late. In a previous study, MERS-CoV ELISA results indicated that the antibody response was highest after 3 weeks from symptom onset [33]. Although no reports have analyzed the duration of antibody presence in MERS patients regardless of symptoms, a recent study of severe acute respiratory syndrome and MERS reported that antibodies in some patients persisted for up to 2-3 years after infection [34,35]. Blood sampling for serologic test in this study was performed on contacts between October and December 2015, while exposure to the confirmed case occurred between May and June 2015 (a gap of 5 months). Thus, a loss of the MERS-CoV antibody titer could have taken place despite actual asymptomatic infections; therefore, the actual rate of asymptomatic infection may be higher than the rate presented in this report.

In conclusion, among 1,610 contacts, only 1 non-healthcare worker who was a patient in a MERS-affected hospital had an asymptomatic MERS-CoV infection. To understand new emerging infectious diseases such as MERS, more intensive epidemiologic research is needed, including an analysis of asymptomatic infections.

## Figures and Tables

**Table 1.** Study subjects and seropositive participants by characteristics of the MERS case they were exposed to, and status of subjects upon MERS exposure in the 2015 Korean outbreak

	Characteristics of exposed MERS case	Status of subjects when they were exposed to MERS^1^							Selection or response rate (%)^2^
		Patient	Family	Visitor	Healthcare worker	Colleague	Unknown	Total	
Quarantined subjects	Spreaders with 5 or more cases	1,141	71	417	558	3	657	2,847	
	Spreaders with 1 to 4 cases	1,067	64	364	198	35	375	2,103	
	Non-spreaders	3,489	321	1,613	1,260	453	2,745	9,881	
	Total	5,697	456	2,394	2,016	491	3,777	14,831	
Selected subjects	Spreaders with 5 or more cases	744	60	254	115	3	212	1,388	48.8
	Spreaders with 1 to 4 cases	191	10	105	10	0	29	345	16.4
	Non-spreaders	626	101	281	184	101	265	1,558	15.8
	Total	1,561	171	640	309	104	506	3,291	22.2
	Selection rate (%)	27.4	37.5	26.7	15.3	21.2	13.4	22.2	
Study participants	Spreaders with 5 or more cases	252	53	152	94	1	0	552	39.8
	Spreaders with 1 to 4 cases	60	4	62	12	0	1	139	40.3
	Non-spreaders	262	109	271	201	50	26	919	59.0
	Total	574	166	485	307	51	27	1,610	48.9
	Response rate (%)	36.8	97.1	75.8	99.4	49.0	5.3	48.9	
Subjects with seropositive ELISA (IFA/PRNT)	Spreaders with 5 or more cases	2 (1)			1 (0)			3 (1)	
	Spreaders with 1 to 4 cases	1 (0)						1 (0)	
	Non-spreaders	3 (0)						3 (0)	
	Total	6 (1)			1 (0)			7 (1)	
	Seropositive rate (%)	1.05 (0.17)			0.33 (–)			0.43 (0.06)	

MERS, Middle East Respiratory Syndrome; ELISA, enzyme-linked immunospecific assay; IFA, immunofluorescence assay; PRNT, plaque reduction neutralization antibody test.

1 Inpatients or outpatients at a MERS-affected hospital, family of people living with or a paid caregiver of the MERS case, visitors of the hospitalized MERS case, healthcare workers employed at a MERS-affected hospital, and colleagues of co-workers of a MERS case.

2 Selection rates varied by characteristics of the MERS case subjects were exposed to (i.e. transmission intensity) and the subjects’ status (i.e. exposure intensity).

**Table 2.** Characteristics of MERS-seropositive subjects by ELISA, IFA, and PRNT in Korea, 2015

Subject ID	Exposure date	Sampling date	Status at exposure	Exposed MERS case ID^1^	Underlying disease	Symptoms after exposure	ELISA		IFA	PRNT
							Ratio	Result		
1	May 15	Nov 1	Patient	#1	Angina	None	0.996	Borderline	Positive	Positive
2	May 16	Nov 2	Patient	#1	None	None	2.078	Positive	Negative	Negative
3	May 15	Nov 2	Healthcare worker	#1	None	Fatigue	1.640	Positive	Negative	Negative
4	Jun 9	Oct 31	Patient	#118	Hypertension	Blurred vision	1.116	Positive	Negative	Negative
5	Jun 4	Nov 8	Patient	#89	Hypertensive heart disease	None	0.916	Borderline	Negative	Negative
6	Jun 5	Nov 9	Patient	#89	Lumbar spinal Fatigue stenosis		1.724	Positive	Negative	Negative
7	Jun 11	Nov 8	Patient	#89	None	Fatigue and	0.985	Borderline	Negative	Negative

MERS, Middle East Respiratory Syndrome; ELISA, enzyme-linked immunospecific assay; IFA, immunofluorescence assay; PRNT, plaque reduction neutralization antibody test.

1 Patient zero (#1) infected 28 cases (super-spreading event transmitting 5 or more cases), case #118 infected 2 cases, and case #89 did not transmit MERS to anyone during the 2015 Korean MERS outbreak.
